# Cutaneous Adverse Events of Immune Checkpoint Inhibitors: A Literature Review

**DOI:** 10.5826/dpc.1101a155

**Published:** 2021-01-29

**Authors:** Zoe Apalla, Chryssoula Papageorgiou, Aimilios Lallas, Florentina Delli, Christina Fotiadou, Christina Kemanetzi, Elizabeth Lazaridou

**Affiliations:** 1 Second Dermatology Department, School of Medicine, Aristotle University of Thessaloniki, Greece; 2 First Dermatology Department, School of Medicine, Aristotle University of Thessaloniki, Greece; 3 State Dermatology Department, Hippokratio General Hospital of Thessaloniki, Greece

**Keywords:** immune checkpoint inhibitors, skin toxicity, adverse effects, nivolumab, pembrolizumab, ipilimumab

## Abstract

Immune checkpoints assist with self-tolerance and minimize collateral tissue damage when immune responses are activated. Although immune checkpoint inhibitors (CPIs) are characterized by a favorable risk/benefit ratio, immune checkpoint blockade has been associated with a new subset of autoimmune-like toxicities, named immune-related adverse events (irAEs). Dermatologic reactions are among the most prevalent irAEs triggered by CPIs. In a majority of cases they are self-limiting and readily manageable. However, it is not uncommon that they result in severe skin involvement and impairment of patients’ quality of life. Awareness of the spectrum of cutaneous irAEs is mandatory for every clinician involved in the management of oncologic patients. The role of the dermatologists is essential because early recognition and appropriate management of skin toxicity may prevent dose modifications and discontinuation of CPIs. The latter is particularly relevant, considering that recent data suggest favorable oncologic response in patients developing irAEs.

## Introduction

### Checkpoint Inhibitor Mode of Action

Checkpoint inhibitors (CPIs) are molecules that increase the endogenous immune response against tumors by blocking receptors responsible for lymphocyte inactivation.

Immune checkpoints include cytotoxic T-lymphocyte antigen 4 (CTLA-4), programmed cell death 1 (PD-1) and programmed cell death ligand 1 (PD-L1). The CTLA-4, PD-1, and PD-L1 pathways mediate immune responses at different levels. CTLA-4 controls the amplitude of immunologic response at early stages of T cell activation, whereas PD-1 and PD-L1 pathways act at later stages, limiting T cell activity in the peripheral tissues. By activating cytotoxic CD4+/CD8+ T cells, immune checkpoint blockade therapy shifts the immune system towards anti-tumor activity [1,2]. Due to their innovative, immune-based mode of action, they entail a new group of adverse effects, which are immune-mediated in their nature. The responsible etiopathogenic mechanism driving cutaneous immune-related adverse events (irAEs) has not been completely elucidated. However, it seems to be connected to the T cell activation, mediated by the blockade of PD-1/PD-L1 and CTLA-4 receptors.

Ipilimumab, an anti-CTLA-4 antibody, was the first licensed CPI after demonstrating improved overall survival in patients with melanoma. The anti-PD-1 monoclonal antibodies block the PD-1 receptor and the anti-PD-L1 monoclonal antibodies block the PD-L1 receptor. In this scenario, blocking with a CPI (either anti-PD-L1, either anti-PD-1) the binding of PD-L1 to PD-1, the T cells are no longer inhibited and therefore the immune response against the tumor is activated [1].

### Implication of CPI Mode of Action in the Development of Cutaneous irAEs

Due to their unique mechanism of action, CPIs result in a new spectrum of adverse events referred to as irAEs. CPIs include anti-PD-1 (nivolumab and pembrolizumab) and anti-CTLA-4 (ipilimumab, tremelimumab) agents, as well as the newly developed anti-PD-L1 agents (atezolizumab, durvalumab, avelumab). Cutaneous irAEs occur in more than one-third of the patients treated with CPIs. Analytically, 50% of individuals receiving ipilimumab and 30%–40% receiving nivolumab or pembrolizumab will experience dermatologic complications [3]. However, severe symptoms of dermal toxicity is reported in <3% cases with anti-PD-1 monotherapy and <5% in combined therapy with anti-CTLA-4 and anti-PD1. In general, CPIs are considered to present an acceptable skin toxicity profile.

### Time of Onset

Cutaneous irAEs may have a delayed onset and prolonged duration, compared to adverse events (AEs) resulting from classic chemotherapy. For most patients, dermatologic toxicity is the earliest occurring irAE [4]. The time of onset of a dermatologic toxicity ranges from a few weeks to several months from treatment initiation, depending on the type of cutaneous irAE.

The relationship between irAEs and dose or time of exposure to CPIs has not been fully elucidated [5]. In this context, clinicians must remain vigilant to the possibility of late onset of irAEs that in some cases may extend to months, or even years, after treatment discontinuation [1,2].

## Classification of Cutaneous irAEs

CPIs can induce a wide variety of skin reactions that represent either a reactivation or deterioration of a preexisting dermatosis or a de novo development. Classification of cutaneous irAEs is still vague and at the moment is mostly based on clinical morphology. In this context, CPI-mediated AEs can be classified into 6 main categories, namely, inflammatory dermatoses, bullous eruptions, pruritus, pigmentary disorders, severe cutaneous adverse reactions (SCARs)/life-threatening drug reactions, and miscellaneous skin manifestations (Table 1). Of note, misclassified cases do exist and they are attributed to various reasons. One of them is that clinicians other than dermatologists that are not sufficiently trained in dermatology are involved in the evaluation and management of skin toxicities. Apart from that, classification systems are vague per se, since there is a lack of histopathological studies that precisely characterize the nature of these rashes. In this context, especially in atypical cases, misclassification is not an unlikely scenario even for expert dermatologist. In such cases, only histopathology can prompt an accurate diagnosis; therefore, if it is not performed, many cases remain misclassified and influence the overall incidence of each skin reaction [6].

The last column in Table 1 reports the frequency of cutaneous reactions to give better guidance to clinicians.

### A. Inflammatory Skin Reactions

**Figure 1:**
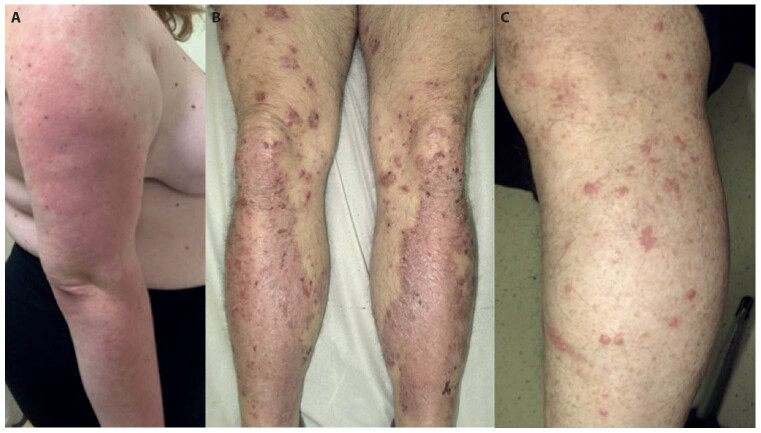
The most common skin toxicities during the course of immunotherapy include (A) maculopapular, (B) psoriasiform and (C) lichenoid eruptions.

Several skin rashes belong to this group, including maculopapular, lichenoid, psoriasiform and eczematous rashes, as well as classical skin irAEs like erythema multiforme (EM), palmoplantar erythrodysesthesia and neutrophilic dermatoses, such as Sweet syndrome [6]. Figure 1 illustrates typical examples of common cutaneous irAEs.

#### 1. Maculopapular (Morbilliform) Rashes

The pruritic maculopapular rash (Figure 1A) is the main representative of this category, as it is the most frequent cutaneous irAE observed with both PD-1/PD-L1 and CTLA-4 inhibitors. Despite its high incidence, the rate of grade ≥ 3 rash is rather uncommon. It mainly involves the trunk, and to a lesser degree the upper extremities, while the face is usually spared. Pruritus, typically developing concomitantly with the rash, or preceding the skin alterations, can be also present as an independent symptom in otherwise normal-appearing skin. The rash usually appears early after treatment initiation in a timeframe that ranges from a few days or weeks (sometimes immediately after the first cycle) to a few months, but delayed eruptions have also been reported. The onset is slightly earlier with ipilimumab or when CPIs are prescribed in combination. It is not uncommon for this nonspecific rash to represent the initial manifestation of other CPI-induced dermatoses, such as lichenoid reactions, psoriasis, Grover disease, bullous pemphigoid or even SCARs. Considering that irAEs represent a newly introduced group of skin reactions, a skin biopsy with histological examination is highly recommended in order to establish the correct diagnosis. Apart from that, close monitoring on a weekly or every 2-week basis facilitates adequate management. Even though histological studies of cutaneous irAEs are scarce in the literature, it seems that the histologic alterations observed in maculopapular rash include eczema-like spongiotic dermatitis and histologic features reminiscent of dermal hypersensitivity reactions [6,7].

In the management of mild (<10% of body surface area [BSA]) or moderate (10%–30% BSA) maculopapular eruption with or without symptoms such as pruritus, burning, tightness, CPI therapy can be maintained. The rash should be treated with topical moisturizing emollients, oral antihistamines for pruritus and medium- to high-potency topical corticosteroids. Systemic prednisone should be preserved for persistent and severe cases (>30% BSA, with or without associated symptoms and limiting self-care, or instrumental activities of daily living). A multidisciplinary team, consisting of oncologists and dermatologists, should decide on the optimal management of patients with severe irAEs. In this scenario, the withholding immunotherapy might be warranted. When skin toxicity is resolved and corticosteroids are reduced at a prednisone dose equivalent to less than 10 mg/day, CPI therapy can be resumed [6–8].

#### 2. Lichenoid Rashes

**Figure 2:**
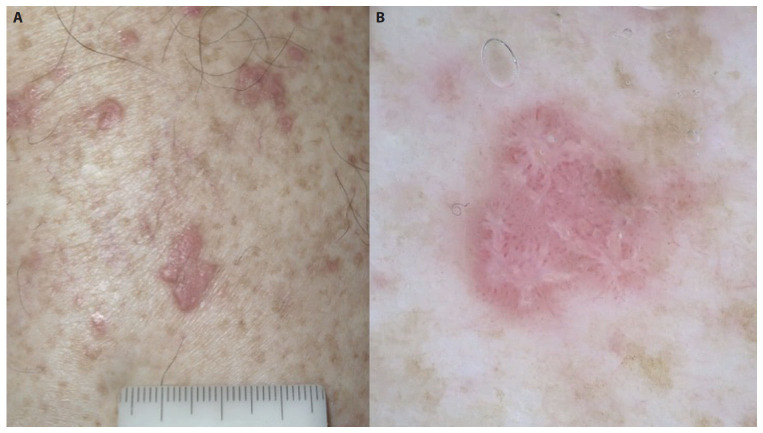
(A) A lichenoid eruption in a patient with Merkel cell carcinoma treated with pembrolizumab. (B) Dermoscopy highlights the Wickham striae, facilitating the diagnosis.

Lichenoid drug eruption (Figure 1C) is another common skin reaction triggered by CPIs. The incidence of lichenoid reactions is probably underreported, and many authors strongly believe that the lichenoid rash is even more frequent than the maculopapular rash, which is also observed in high rates among individuals treated with CPIs. In doubtful cases, dermoscopy might prove helpful, since it highlights the Wickham striae, facilitating the diagnosis of lichenoid eruptions (Figure 2, A and B). The responsible immunologic mechanism is not completely elucidated. However, it is assumed that the inhibition of PD-1, PD-L1, or CTLA-4 results in impaired T cell homeostasis in the skin, thus facilitating such cytotoxic inflammatory reactions. The onset of lichenoid dermatologic toxicity to CPIs usually develops several weeks to months after treatment initiation. The lesions may resemble those seen in typical lichen planus or may be hypertrophic, papulosquamous, or even bullous. Sites of predilection are the trunk and extremities. Pruritus is frequent and in some instances can be so severe profoundly diminishing patients’ quality of life [6,7]. Oral and genital mucosa may also be involved [9]. Severe forms need to be differentiated from other severe cutaneous toxicities, such as Stevens-Johnson syndrome/toxic epidermal necrolysis (SJS/TEN). In consideration of the aforementioned morphologic diversity, histopathologic examination is of paramount importance to establishing a definite diagnosis. A dense band-like lymphohistiocytic infiltrate along the dermal–epidermal junction, with patchy-to-florid vacuolar interface dermatitis and basilar/suprabasilar apoptotic keratinocytes compose the histopathologic pattern of CPI-mediated lichenoid rash [10].

Therapeutic management includes topical steroids and, rarely, oral corticosteroids, phototherapy, or acitretin. CPI treatment is usually maintained [6–8].

#### 3. Psoriasiform Rashes

**Figure 3:**
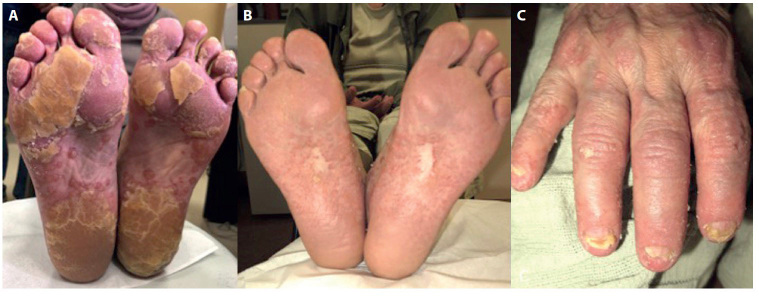
(A) A patient with non-small cell lung carcinoma who was treated with a combination of nivolumab and ipilimumab developed palmoplantar pustulosis after the third cycle. (B) Significant improvement of the skin after 2 months of treatment. (C) However, the patient experienced severe psoriatic arthritis and was switched to apremilast.

CPIs may trigger a psoriasiform rash, or may exacerbate a preexisting psoriasis (Figure 3, A-C). It is well known that psoriasis is a chronic, immune-mediated, inflammatory skin disease [11,12]. The pathogenetic mechanism responsible for psoriasis in patients treated with CPIs has not yet been completely elucidated. However, it is deemed to be immune-mediated, resulting by the T-cell activation of cytotoxic CD4+/CD8+ [13,14,15].

Well-demarcated, erythematous, scaly papules and plaques, reminiscent of classic plaque psoriasis, is the commonest presentation. In addition, guttate, pustular, and inverse psoriasis have also been reported [16,17]. Importantly, exacerbation or de-novo occurrence of psoriatic arthritis (Figure 3C), with or without skin psoriasis, has also been described [18]. The histopathologic findings are similar to those seen in classic plaque psoriatic cases.

Topical treatment does not differ from the treatment of classic psoriasis and includes vitamin D analogues and topical steroids. Among the systemic treatments, retinoids, UVB therapy, apremilast and methotrexate are preferable. With the exception of erythrodermic and generalized pustular psoriasis, CPI therapy is usually maintained. Early recognition and adequate management of CPI-mediated psoriasis are challenging, especially considering the lack of evidence-based guidelines. In any case, a multidisciplinary approach is necessary.

#### 4. Eczematous Rashes

Eczematous rashes are also common in patients treated with CPIs. They are characterized by pruritic, erythematous, scaly, or crusted macules and papules that may coalesce into plaques. Diverse clinical presentation, including localized or generalized patches/plaques of classic dermatitis, as well as nummular, dyshidrotic and asteatotic eczema may be observed. The time of onset ranges from 4–18 months after treatment initiation.

#### 5. Neutrophilic Dermatoses

The spectrum of neutrophilic dermatoses secondary to CPIs includes Sweet syndrome, pustular eruptions, bullous lupus erythematosus, and pyoderma gangrenosum. They are considered rare, with only few cases reported [19]. The onset of neutrophilic dermatoses seems to be relatively delayed, ranging from weeks to months. Interestingly, most of the cases of Sweet syndrome and all cases of pyoderma gangrenosum reported in the literature were induced by ipilimumab (anti-CTLA-4) [20].

### B. Bullous Eruptions

**Figure 4:**
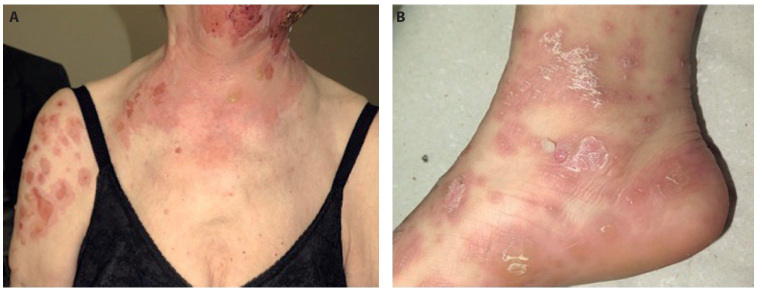
Uncommon skin toxicities include bullous disorders like (A) bullous pemphigoid and (B) bullous lichen planus.

Compared with other dermatoses, autoimmune bullous diseases in association with immunotherapy are less common and they are scarcely reported in the literature (Figure 4). Interestingly, the risk of a bullous eruption seems to be higher when treated with anti-PD-1 or anti-PD-L1 rather than anti-CTLA-4. In the analysis of a database including 853 patients receiving anti-PD-1/PD-L1, the rate of bullous skin toxicity was about 1% in the studied population [21]. Of note, most of them developed bullous pemphigoid (BP). In addition, a case of bullous lichenoid dermatitis and a case of linear IgA bullous dermatosis were also reported. In another retrospective analysis, the incidence of bullous disorders was higher (8%) and all cases were secondary to anti-PD1/PD-L1 therapy [22]. The clinical presentation of bullous disorders includes pruritus, tense vesicles/bullae on the trunk and extremities as well as oral erosions, and urticarial lesions. The eruption may appear either early after initiation of therapy or only after several months of treatment. Interestingly, the anti-PD-1/PD-L1-induced BP may persist for several months after discontinuation of the treatment in contrast to the classic BP that usually disappears immediately after discontinuation of the causative agent [23]. The diagnosis is established by the combination of clinical features, histopathologic and immunohistochemical findings, and direct and indirect immunofluorescence.

In a literature review of BP associated with PD-1 and PD-L1 inhibitors, including all the publications up to July 2017, the authors identified 21 cases [24]. Twelve of them experienced nonspecific cutaneous features with pruritus before or along with the clinical appearance of the blisters, and 16/21 necessitated discontinuation of PD-1/PD-L1 inhibitor due to BP. The latter was mainly managed with diverse doses of systemic steroids that were used either as monotherapy or in combination with other systemic agents, including antihistamines, doxycycline, niacinamide, methotrexate, omalizumab and rituximab. Additionally, topical treatment was applied in most cases.

As for the treatment strategy of immunobullous dermatoses, according to the existing guidelines [6–8], when symptoms are mild (blisters covering <10% BSA), high-potency topical steroids may be applied to the affected areas and immunotherapy should be withheld. In moderate cases (blisters covering 10%–30% BSA; painful blisters limiting instrumental activities of daily living), prednisone/methylprednisolone 0.5–1 mg/kg per day should be added. In severe cases (blisters covering >30% BSA limiting instrumental activities of daily living), immunotherapy should be discontinued and hospitalization should be considered, along with dermatologic and other appropriate consulting services (eg, ophthalmology; urology; gynecology; etc) if needed. In these cases, prednisone/methylprednisolone 1–2 mg/kg per day should be administered, and if no improvement is noted after 3 days, adding rituximab should be considered. General recommendations include topical wound care with petrolatum ointments and gauzes over erosions and avoidance of sun exposure. Total body skin examination that includes all skin surfaces and mucous membranes is highly recommended. In addition, lymphadenopathy, facial or distal extremity swelling, and Nikolsky sign should be assessed, as they may be signs of drug-induced hypersensitivity syndrome (DIHS), DRESS or SJS and TEN. In any case, patients should be closely monitored and close collaboration between dermatologists and oncologists is essential for the optimal treatment decision [25].

Whether the appearance of BP indicates a favorable response to immunotherapy or not remains unknown. However, in a recently published study, the authors reported improved outcomes in the group of patients that developed BP compared to controls [26].

### C. Pruritus

Pruritus is one of the most common irAEs during therapy with CPIs. The incidence ranges from 14% to 21% in patients receiving anti-PD-1, which is higher compared to anti-CTLA-4. Combination treatment with nivolumab and ipilimumab further increases the occurrence of pruritus. However, the incidence of severe cases is low [27–31]. The lower incidence of pruritus is recorded with anti-PD-L1 treatment [32]. Pruritus may develop either per se or may be associated with skin rashes, irritation and xerosis, and early after immunotherapy initiation. In any case, it can highly aggravate a patient’s quality of life and psychological status, making the need for relief therapy mandatory. Guidelines for management of mild or moderate pruritus recommend oral antihistamines and topical steroids of mild and high potency, while prednisone/methylprednisolone and GABA agonists are preserved for more severe cases. In the end, aprepitant or omalizumab may be considered for refractory cases [8,33].

### D. Pigmentary Disorders

**Figure 5:**
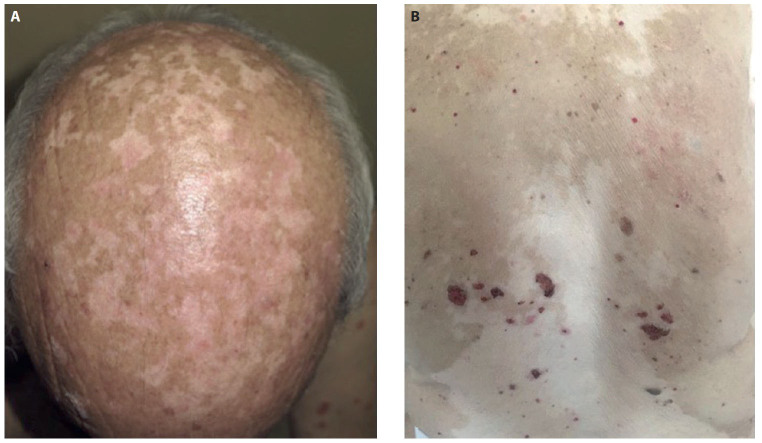
Concomitant development of more than one skin toxicity is not unusual. The patient illustrated in (A) experienced vitiligo and psoriasis (see Figure 1B) and the patient in (B) experienced vitiligo and bullous pemphigoid, both during treatment with nivolumab for advanced melanoma.

Vitiligo represents another common cutaneous irAE, particularly reported in melanoma patients treated with CPIs. Only exceptional cases have been described in patients with tumors other than melanoma [34,35]. The incidence of vitiligo ranges from 7.5% to 25% in patients treated with anti-PD-1, and it is less frequent with the use of anti-CTLA-4 agents. The exact mechanism of vitiligo remains unclear. However, taking into account its strong association with melanoma, an autoimmune mechanism has been hypothesized. Specifically, melanoma shares common antigens with healthy melanocytes that are involved in melanin synthesis. Therefore, the antibodies directed to these melanoma-associated antigens also target healthy melanocytes. This, along with the cytotoxic T cell-mediated response induced by immunotherapy, finally result in depigmentation [36]. Vitiligo develops progressively after several months of immunotherapy and in most cases does not resolve even after treatment discontinuation. The lesions are usually distributed bilaterally and tend to be generalized. However, focal or localized depigmentation, sometimes surrounding cutaneous metastases, may be also observed [37]. Fading and disappearance of nevi and other pigmented lesions, such as solar lentigines and seborrheic keratosis, can also occur [38]. Interestingly, the development of new nevi has also been described [39]. Hair involvement, with whitening of the eyelashes, eyebrows, or scalp hair is not unusual. Although vitiligo may affect the patient’s psychological status, other than sun protection, it requires no treatment. Importantly, the coexistence of vitiligo with other cutaneous irAEs is not uncommon (Figure 5).

Recently published data show that the development of vitiligo-like depigmentation suggests a favorable response to treatment in patients receiving pembrolizumab or nivolumab [36,40–43]. Specifically, it has been associated with prolonged progression-free survival and overall survival and could probably serve as a positive prognostic factor of the oncologic outcome. However, further studies are required to validate these results.

### E. Severe Cutaneous Adverse Reactions (SCARs)/Life-Threatening Drug Reactions

SCARs encompass DRESS/DIHS, SJS, TEN, and acute generalized exanthematous pustulosis [44]. SCARs are scarcely reported with CPIs [45–51]. Considering that a maculopapular or a nonspecific rash may precede life-threatening reactions, dermatologic consultation and possibly a skin biopsy is recommended.

In case of a SCAR, CPI therapy must be withheld or permanently discontinued and hospitalization maybe needed for appropriate management [7,8].

### F. Miscellaneous Skin Manifestations

This category encompasses a series of rare skin complications that may occur during CPI therapy.

Grover disease may present as pruritic erythematous papules or keratotic papules, papulovesicles, and vesicles [52] The lesions may be distributed on the trunk and chest or may be more diffuse, usually appearing early after the CPIs initiation and may last several weeks or months after their discontinuation [53,54]. A skin biopsy is mandatory for diagnostic purposes.

Rare autoimmune disorders, such as vasculitis, dermatomyositis and Sjögren syndrome during anti-PD-1/anti-PD-L1 and anti-CTLA-4 therapy have been sporadically reported [55–59]. In such cases, laboratory tests should be performed and systemic involvement should be excluded.

Furthermore, de-novo development or exacerbation of preexisting sarcoidosis, urticaria, rocasea, or prurigo simplex/nodularis, may occur [22,60,61]. Sarcoidosis is not uncommon and its clinical presentation may vary from papules or plaques to erythema nodosum, with or without pulmonary or other organ involvement [62–65]. Systemic or topical steroids may be applied for the treatment of the skin lesions.

In the end, oral mucosa alterations and nonspecific nail and hair alterations, like onychodystrophy, paronychia, alopecia and dysgeusia can be observed. Alopecia is usually non-scarring, is mainly of alopecia areata type, and can be partial or diffuse. The underlying mechanism involves an immune attack operated by cytotoxic CD8+ cells on the hair bulb [66].

## Treatment Strategy

In most cases, cutaneous irAEs are mild (grade 1, 2) and usually manageable with topical treatment, including steroids, calcineurin inhibitors and phototherapy. Skin toxicity grade ≥ 3, usually requires systemic immunomodulating and immunosuppressive drugs. Systemic steroids, though highly efficacious and widely used in cutaneous drug reactions, raise practical difficulties when used in high doses, since they may impede the action of immunotherapy. In this context, involvement of qualified dermatologists that may apply organ-specific therapeutic modalities and avoiding immunosuppressive agents is crucial for immunotherapy survival. Furthermore, considering that the impact of various immunomodulating drugs on the anti-tumor effect of immunotherapy has not been fully elucidated, close collaboration between oncologists and dermatologists is mandatory for the optimization of treatment strategy (eg, doses, dosage modifications, discontinuation or not of immunotherapy) that will finally benefit the patient. In a recent commentary, the authors proposed an algorithm that could serve as a guide for treatment decisions of both dermatologists and oncologists [67].

## Conclusions

In conclusion, the immune-based mechanism of CPIs results in a novel toxicity profile that differs from the one observed by traditional cytotoxic therapies. Overall, the CPI profile is favorable when compared with standard anti-cancer agents, such as chemotherapy and targeted therapy. However, close monitoring for symptoms of irAEs when prescribing CPIs is mandatory. The most common cutaneous AEs include maculopapular, lichenoid, psoriasiform and eczematous skin rashes, pruritus, and pigmentary disorders. Skin toxicities usually occur early in the course of treatment, and in general, tend to be less severe during therapy with anti-PD-1/anti-PD-L1 agents compared to CTLA-4 inhibitors or combinations. In all cases, early recognition of cutaneous immunotherapy-driven AEs is of paramount importance for the patient, as it allows adequate control of the toxicity and increased survival of immunotherapy. In this context, a multidisciplinary team, including specialized dermatologists and oncologists, is desirable for optimal management. Finally, large-scale studies will elucidate certain aspects in cutaneous irAEs pathogenesis, prognostic factors for their development, their prognostic value for the oncologic outcome, and optimal management strategies. Furthermore, the impact of systematic therapies (eg, corticosteroids and immunosuppressive drugs) that are usually used to treat severe skin reactions on the anti-tumor effect of immunotherapy is largely unknown and requires further research.

## Figures and Tables

**Table 1:** Classification of Skin Toxicities Induced by Checkpoint Inhibitors

Category	Skin Disorder	Frequency

Skin rashes/inflammatory dermatitis	Maculopapular Lichenoid Psoriasiform Eczematous Neutrophilic dermatoses	Frequent Frequent Frequent Frequent Rare
Bullous eruptions	Bullous pemphigoid, bullous lichen, lichen pemphigoides, linear IgA bullous dermatosis, bullous drug eruption	Rare
Pruritus	Isolated or in association with skin rashes	Frequent
Pigmentary disorders	Vitiligo Regression of melanocytic nevi and other pigmented lesions	Frequent Less frequent (pigmentary disorders are mainly seen among melanoma patients while it is rare in other tumors)
Severe cutaneous adverse reactions/life-threatening drug reactions	Drug reaction with eosinophilia and systemic symptoms (DRESS)/drug-induced hypersensitivity syndrome (DIHS) Steven-Johnson syndrome (SJS) Toxic epidermal necrolysis (TEN) Acute generalized exanthematous pustulosis (AGEP)	Rare
Miscellaneous	Grover disease Vasculitis Dermatomyositis Sjögren syndrome Sarcoidosis Urticaria Acneiform/papulopustular rocasea Prurigo simplex/nodularis Nail, hair, oral mucosa	Rare Rare Rare Rare Less frequent Rare Rare Rare Less frequent
